#  The habits of highly effective phages: population dynamics as a framework for identifying therapeutic phages

**DOI:** 10.3389/fmicb.2014.00618

**Published:** 2014-11-18

**Authors:** James J. Bull, Jason J. Gill

**Affiliations:** ^1^Institute for Cellular and Molecular Biology, University of Texas, Austin, TXUSA; ^2^Center for Computational Biology and Bioinformatics, University of Texas, Austin, TXUSA; ^3^Department of Integrative Biology, University of Texas, Austin, TXUSA; ^4^Department of Animal Science, Texas A&M University, College Station, TXUSA; ^5^Center for Phage Technology, Texas A&M University, College Station, TXUSA

**Keywords:** bacteriophage, phage therapy, mathematical model, population dynamics, bacterial infections

## Abstract

The use of bacteriophages as antibacterial agents is being actively researched on a global scale. Typically, the phages used are isolated from the wild by plating on the bacteria of interest, and a far larger set of candidate phages is often available than can be used in any application. When an excess of phages is available, how should the best phages be identified? Here we consider phage-bacterial population dynamics as a basis for evaluating and predicting phage success. A central question is whether the innate dynamical properties of phages are the determinants of success, or instead, whether extrinsic, indirect effects can be responsible. We address the dynamical perspective, motivated in part by the absence of dynamics in previously suggested principles of phage therapy. Current mathematical models of bacterial-phage dynamics do not capture the realities of *in vivo* dynamics, nor is this likely to change, but they do give insight to qualitative properties that may be generalizable. In particular, phage adsorption rate may be critical to treatment success, so understanding the effects of the *in vivo* environment on host availability may allow prediction of useful phages prior to *in vivo* experimentation. Principles for predicting efficacy may be derived by developing a greater understanding of the *in vivo* system, or such principles could be determined empirically by comparing phages with known differences in their dynamic properties. The comparative approach promises to be a powerful method of discovering the key to phage success. We offer five recommendations for future study: (i) compare phages differing in treatment efficacy to identify the phage properties associated with success, (ii) assay dynamics *in vivo*, (iii) understand mechanisms of bacterial escape from phages, (iv) test phages in model infections that are relevant to the intended clinical applications, and (v) develop new classes of models for phage growth in spatially heterogeneous environments.

## INTRODUCTION

Phage therapy, long practiced in some countries, is enjoying a rebirth in Western medicine (Gill, 2010; Abedon et al., 2011; Gill and Young, 2011; Ryan et al., 2011; Tsonos et al., 2013a). As currently practiced, phage therapy seems to be a largely idiosyncratic discipline, in that there are few fundamental principles that can be defended or refuted with experimental validation, other than the obvious ones of using phages that infect the offending bacteria and applying a broad spectrum of phages simultaneously. Significant thought has been given to the rational design of phage-based therapeutics (Gill and Hyman, 2010; Goodridge, 2010), but such considerations are limited by our understanding of phage biology and the *in vivo* realities of antimicrobial treatment. To date, most of what can be said about the rational choice of a therapeutic phage is related to its host range and the possibility of phage resistance, and some practical considerations such as phage stability and ease of production. Other phage characteristics, such as high adsorption rates, *in vivo* persistence, or high fecundity (short latent periods and large burst sizes) are presumed to be beneficial but with little understanding as to what specific parameter values are compatible with treatment success.

It may be that the only property required for efficacy is a phage’s ability to form plaques on a lawn of the infecting bacterial strain. If so, there will be little need for principles beyond the determination of phage host ranges before deployment as therapeutics. Ability to infect the target strain is obviously a necessary condition, but it need not be sufficient, and as the field has progressed, it has become apparent that not all phages are equivalent in treating acute infections (Smith and Huggins, 1982; Henry et al., 2013), and many studies of chronic infections reveal that bacteria are not eliminated by phages, even though clinical symptoms are often reduced (Scott et al., 2007; Hurley et al., 2008; El-Shibiny et al., 2009; Tsonos et al., 2013b); treatment of plant infections has also revealed efficacy differences among phages (Balogh et al., 2010). Even more problematic, the literature may be biased toward phage therapy successes (Tsonos et al., 2013a), obscuring the extent to which the type of phage or treatment matters to the outcome.

Even when phages work well at curing infections, little is known about the phage-bacterial dynamic or what is needed for success. This poor understanding complicates the development of phage therapy as a science. There are no broad spectrum phages to rival antibiotics: phage host ranges are too narrow, typically limited to a subset of strains within a bacterial species. Although a narrow host range is often considered an advantage of phage therapy over antibiotics, it does mean that many different phages will be needed, and thus must be tested, to provide treatments effective against infections caused by different pathogens. The hope is that there may be ‘broad spectrum properties’ of phages that can be employed in choosing successful phages *a priori* with a minimum of experimental failure. To do so requires understanding what distinguishes successful phages from unsuccessful ones, and why.

This paper is thus motivated by two premises. First, for many types of infections, not all phages are equally effective at enabling recovery. Second, we propose that there are properties of efficacious phages that will generalize across different infections so that, when those properties are known, they can be used to select phages suitable for treatment. While we review several properties that have been proposed, this paper is equally about the search for principles and methods that might be used in that search. As phage control of bacteria is intrinsically one of population dynamics, we apply that perspective. The paper combines a review of previous work with a synthesis of existing models and observations and offers recommendations for future studies.

## PREVIOUSLY SUGGESTED PRINCIPLES

We begin with a list of principles that have been proposed to benefit treatment (**Table 1**). Rationales behind these principles have been discussed elsewhere and so will not be covered in depth here (references to previous works are provided in **Table 1**). While it is obvious that a phage must be able to infect a bacterial strain to be useful for treatment (thus it is not listed here), the other principles have been practiced to varying degrees, with the majority of phage therapy studies focusing on the first two or three criteria listed in **Table 1**. A principle may be widely acknowledged without being widely implemented (e.g., avoidance of temperate phages is widely appreciated, but phages are rarely rigorously confirmed to be obligately lytic before use).

**Table 1:** Suggested principles in the choice of phages for therapy.

	Principle	Benefits	Drawbacks	Reference
1	Broad host range	Reduces need to diagnose infecting strains; may simultaneously attack multiple strains of a single pathogen	Broad host range may conflict with other optimal phage characteristics	Balogh et al. (2010), Gill (2010)
2	Phage mixtures targeting different host receptors	Development of resistance to phage less likely as mutants resistant to one phage remain sensitive to others in the mixture	Greater development time, may limit the repertoire of phage available for some bacteria	Balogh et al. (2010), Gill (2010), Chan and Abedon (2012), Chan et al. (2013)
3	Non-temperate	Avoids lysogeny as an easy form of bacterial insensitivity; avoids pathogenicity genes commonly found in temperate phages	Limits the repertoire of phage available for some bacteria	Gill and Young (2011)
4	Ability to clear liquid cultures	Simple *in vitro* assay	Applicability to *in vivo* success unknown	Smith and Huggins (1982), Henry et al. (2013)
5	Non-lysing phages	Cells are killed without releasing toxins	Phage do not amplify, so huge numbers of phage must be inoculated	Matsuda et al. (2005)
6	Target surface virulence determinants or otherwise impose high cost of resistance	Difficult bacterial escape; resistant cells become unfit/avirulent	Limits the repertoire of phage available	Capparelli et al. (2010a,b), Viertel et al. (2014), this paper
7	Tailspike de-polymerase	Acapsular bacterial mutants are often avirulent (see item 6); unassembled tailspikes released at lysis as free enzyme, digest capsule of nearby cells and expose them to immune system	Possibly limited host range of such phage, limits the repertoire of phage available for some bacteria	Francis et al. (1934), Sutherland (1995), Mushtaq et al. (2004), Malnoy et al. (2005), Bull et al. (2010)
8	Non-transducing	Will not mobilize pathogenicity or antibiotic resistance determinants	Limits the repertoire of phage available for some bacteria	Gill (2010)
9	Slow *in vivo* virion decay rate	Increases phage longevity in the animal host, increasing probability of phage encountering bacteria	None obvious, may allow for faster development of acquired immunity to phage	Merril et al. (1996), Balogh et al. (2010)

Advantages of several of these principles are not obviously relevant across all therapeutic applications. The ability to clear liquid cultures or other indicators of *in vitro* growth have been reported as a positive indicator of success in some cases (Smith and Huggins, 1982; Henry et al., 2013), but not in others (Balogh et al., 2010; Tsonos et al., 2013b); without a mechanistic link between *in vivo* and *in vitro* growth, this assay is not a guarantee of success. Many of the principles proposed have no obvious downside beyond practical considerations of finding phages with the desired characteristics, but how crucial each characteristic is for treatment success is far from being resolved. For example, phage targeting of surface virulence determinants is probably an advantage, but there are many reports of treatment success using phages that do not seem to specifically target receptors critical to virulence (e.g., Merril et al., 1996; Matsuzaki et al., 2003; Atterbury et al., 2007) so this criterion is apparently not an absolute requirement for success.

These suggested principles are surprisingly devoid of dynamical considerations, other than the obvious one that the phage must be able to grow on the infecting strain. Principle 4 (the ability to clear liquid cultures) is the closest exception, although the outcome of this type of assay is the product of several individual dynamic parameters. This omission of suggested dynamical principles leads to an important question, considered next.

## THE CENTRAL QUESTION: HOW IMPORTANT ARE DYNAMICS TO TREATMENT SUCCESS?

In the search for successful phages, it is important to know whether a phage’s success is tied closely to its intrinsic dynamical properties: what are the relationships between phage replication rates, the rate of phage killing of the bacterial population and the probability of treatment success? It is probably generally true that phage success requires a minimum level of growth on the target population, but beyond that minimal requirement, does incrementally better phage growth lead to enhanced success? Indeed, the paucity of population dynamics principles in **Table 1** raises the possibility that dynamic principles are not important to phage therapy success (alternatively, dynamics may have not been studied). Determining whether and under what conditions phage growth and host killing correlate with success – and how they contribute – should be a goal of the phage therapy enterprise. This paper thus considers what we know about the relationship between phage dynamics and bacterial control as well as how that relationship may be assessed.

### MATHEMATICAL MODELS OF DYNAMICS

On a qualitative level, we know that phage growth depends on bacterial numbers. Yet phage growth reduces bacterial numbers, and this bacterial decline feeds back by reducing future phage numbers. It is thus easily appreciated that the quantitative dynamics of the phage-host interaction are too complicated for intuition, motivating the use of mathematical models. Mathematical models of dynamics can potentially serve multiple purposes, from merely guiding intuition, to yielding qualitative results that can be evaluated empirically, to providing a quantitative fit to empirical data. We begin by reviewing the models that have been proposed and how they may be used to understand dynamics.

The long history of pharmacodynamic and kinetic studies of antibiotics and bacteria inspired parallel models of phage-bacterial infection dynamics with the specific goal of understanding *in vivo* processes; the efforts are still few (Levin and Bull, 1996, 2004; Payne and Jansen, 2001, 2003; Weld et al., 2004; Bull and Regoes, 2006; Abedon, 2011, 2012). Levin and Bull (2004, 1996) addressed dynamics at high bacterial densities, when phages proliferate, whereas Payne and Jansen (2001, 2003) emphasized dynamics when bacterial densities are low or when phage may otherwise be unable to maintain themselves. Those models of *in vivo* phage dynamics are little more than standard models of *in vitro* dynamics, with the usual assumptions of mass action (diffusion in liquid) and spatial plus temporal homogeneity of parameters and processes (see Levin and Bull, 1996; Weld et al., 2004; Bull and Regoes, 2006; Abedon, 2012 for some exceptions). Phage growth in liquid is determined by three parameters, the adsorption rate constant, burst size, and lysis time or latent period (**Table 2**). A homogeneous culture of one phage type (density *P*) attacking one bacterial type (density *B*) is described by two differential equations:

(1) dB/dt=αB−kPB−δB

(2) $$dP/dt=bkP_{L}B_{L}-kPB-\delta P$$

(e.g., Adams, 1959; Campbell, 1961), with notation given in **Table 2**. *dB/dt* and *dP/dt* are rates of change with time of bacterial and phage densities; variables subscripted with *L* indicate the value of that variable *L* minutes in the past. Payne and Jansen (2001) added terms for time-dependent decay rates due to host response but essentially ignored those terms in their analyses, reflecting the difficulty in empirical determination of these values.

**Table 2:** Parameter definitions.

Notation	Description	Units
α	Growth rate of uninfected bacteria	/min
b	Burst size: number of phage progeny released at lysis	PFU
k	Phage adsorption rate constant	mL/min
L	Time to host cell lysis after phage infection	min
δ	Death or removal rate	/min

The chief unintuitive process in these equations is adsorption (attachment of the virion to the bacterium), which proceeds according to second-order reaction kinetics. At a mathematical level, adsorption combines the two processes of virion attachment to the cell and actual infection of the cell; any delay between phage attachment and genome entry is trivially subsumed into lysis time. The adsorption rate constant, *k*, itself combines two processes: (i) the rate of chance encounter by diffusion of a single phage particle with a single bacterium in 1 mL of liquid with (ii) the rate at which the phage initiates an infection (adsorbs) given an encounter. Because of (i), empirical values of *k* are always low – 10^-8^ mL/min or less – reflecting the low rate of chance encounter. Values of *k* much below this approximate limit typically stem from low rates of infection given an encounter. Detailed empirical analyses suggest that infection often follows a 2-step process of reversible adsorption followed by irreversible adsorption (Adams, 1959; Moldovan et al., 2007); the effect of this complication on dynamics has not been modeled but is likely accommodated to a suitable approximation with the standard model. The interrelationships between phage density, bacterial density and *k* will be discussed only in general terms here, as they have been examined extensively elsewhere (e.g., Levin and Bull, 1996; Payne and Jansen, 2001; Gill, 2008; Abedon and Thomas-Abedon, 2010; Abedon, 2011).

By virtue of the deterministic nature of the equations, bacteria are never strictly eliminated in these models – even if the density drops below 1/unit volume, phage are modeled as if present in infinite volumes. Furthermore, phage parameters are constant, so any phage that increases initially will ultimately outgrow bacteria in this model, leading either to a joint equilibrium of phage and bacteria or continual oscillations (Levin et al., 1977). There is thus no intrinsic property of the dynamical equilibrium that can be used to infer host survival or death. Host mortality has been equated with bacterial density reaching a threshold at any point in the infection (Levin and Bull, 1996), or with persistence of the bacterial population beyond a set period of time (Payne and Jansen, 2001). By this criterion of treatment success, faster growth of a phage – better phage fecundity per unit time – always increases treatment superiority. Thus, if the model indeed represents reality, we would readily conclude that phage dynamics directly determine treatment success.

### THE MODELS DO NOT QUANTITATIVELY DESCRIBE REAL INFECTIONS

There are two serious difficulties in using parameterized models as quantitative predictors of real infections. Most importantly, the models are poor characterizations of infections – they neglect inhomogeneous diffusion, host immunity, and time-varying and space-varying parameter values (e.g., Payne and Jansen, 2003). As noted above, the fate of the host is imposed artificially. Some of these omissions can be incorporated easily, but many cannot. Thus even if one could parameterize the models with average parameter values, model utility is dubious. Second, parameterizing even the simple models *in vivo* can be a hopeless task. Weld et al. (2004) showed equations of the sort described above were unable to predict the quantitative interactions between phage T4 and *Escherichia coli* in the mouse gut. Thus the models are more appropriately used for qualitative behaviors that we may hope apply broadly.

The extent of model incompleteness is easily comprehended by considering just the specific empirical realm of phage access and delivery to bacteria. Phages may be applied *in vivo* at very high doses, with MOIs of ten or more based on total bacterial counts of infected tissue. Yet it is rarely if ever known how many of these phage actually reach a bacterium; that is, the *in vivo* values of *P* and *k* are not known and are probably highly heterogeneous within the same animal. Several factors may reduce the number of phage encountering hosts *in vivo*, including partial or complete spatial refuges and rapid clearance of phage by passive or active immunity, or exposure to UV light in the case of plant applications (Balogh et al., 2010). Physical properties of the virion such as surface charge, hydrophilicity, and physical dimensions may affect its ability to penetrate physical refuges, and thus affect the functional *in vivo* adsorption rate. It has been observed that bacterial growth status can have a significant effect on all three of the major model parameters: starved *E. coli* cells exhibit lower adsorption rates, longer latent periods, and reduced burst sizes when exposed to phage T4 than cells grown in rich medium (Hadas et al., 1997). Thus the physiology of the bacterial cells is an important consideration when attempting to predict the efficacy of a phage treatment. Phage have also been shown to interact with the *in vivo* environment, such as with mucus present in the human intestine (Barr et al., 2013). In the context of phage treatment of gastrointestinal infections, this phenomenon could prove to be a barrier to exogenously introduced phage by retarding phage diffusion, or could be a benefit by maintaining high local concentrations of phage at the mucosal surface. These complexities provide a mere glimpse of the challenges in developing accurate models of infection dynamics.

### EXISTING MODELS DISPLAY UNINTUITIVE BEHAVIORS THAT MAY QUALITATIVELY DESCRIBE INFECTIONS

Although existing models are not accurate descriptors of *in vivo* systems, they can be useful in identifying dynamical behaviors that are potentially observable in infections at a qualitative level. There are of course obvious inferences from the models – larger burst and adsorption rates or shorter lysis times are always beneficial when considered in isolation. Also, the dynamics of bacterial killing depend both on the inoculum dose and subsequent phage replication; a sufficiently large phage inoculum can reduce bacterial numbers below a threshold without any phage reproduction at all – the ‘passive’ therapy of Payne and Jansen (2001); use of large inoculum doses may help in many applications where bacterial densities are low or phage exhibit poor growth (Bull and Regoes, 2006).

Some dynamic properties are less obvious. We describe three examples that may facilitate choosing phages for therapy and diagnosing the causes of failure.

#### Bacteria and phage can be maintained at high densities when adsorption rate is low

**FIGURE 1:**
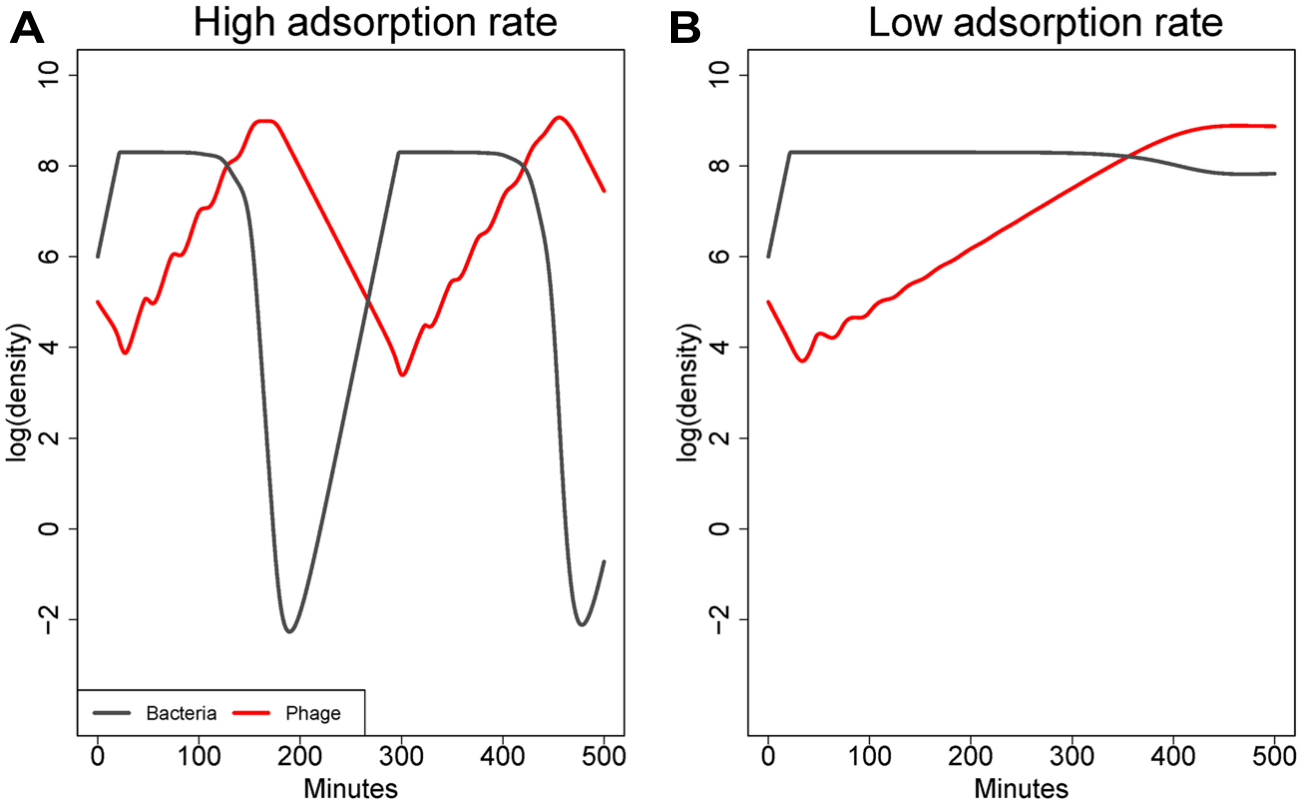
**Co-dynamics of bacteria and phage in a model with a constant washout rate. (A)** shows dynamics with an adsorption rate of 10^-9^ mL/min, **(B)** with an adsorption rate of 10^-10^ mL/min. Parameters are otherwise the same between **(A)** and **(B)**. The higher adsorption leads to better killing of the cells. As is typical in these models when adsorption rate is high, densities fluctuate because phage decline (due to washout) after cell density has been depressed, and once phage density declines, bacterial densities rebound until phage catch up. However, with a mere 10-fold decline in adsorption rate, phage can grow but fail to depress bacterial densities substantially. The model is based on differential equations similar to those in Bull et al. (2014).

As illustrated in **Figure 1**, the adsorption rate constant (*k*) has a major effect on the equilibrium densities of both bacteria and phage (at least in systems with constant flow or death rates; Levin et al., 1977). High values of *k* – those near 10^-8^ mL/min or higher – enable phage to suppress bacterial densities to low levels. Values of *k* that are 2–3 logs lower relax phage control of bacteria, resulting in an equilibrium at which both bacteria and phage are abundant (Levin et al., 1977). Although those bacteria are technically sensitive to phage, and phage do indeed replicate on the bacteria, bacterial densities are only minimally impacted by phage at the time scales typically under consideration. Note that this process is fundamentally different than the failure of phage to control bacteria due to low bacterial density identified by Payne and Jansen (2001). In an infection, the high phage density might give the impression that phage were having a large effect, but the high bacterial density would belie that interpretation. Thus this model property could help resolve a common empirical paradox.

#### Adsorption rates may not be homogeneous in a bacterial population

**FIGURE 2:**
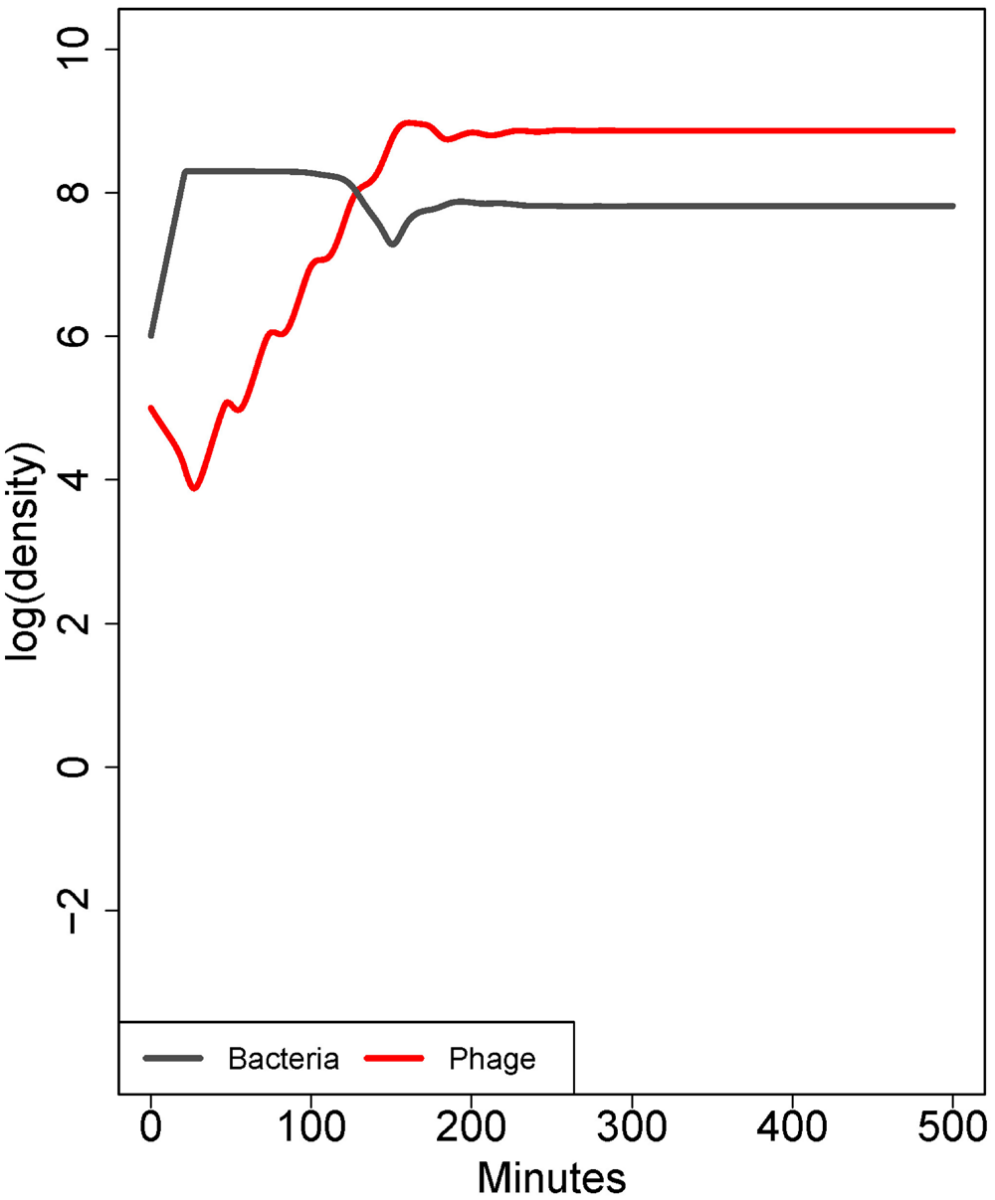
**Co-dynamics of bacteria and phage.** In contrast to **Figure 1**, here the population consists of a mix of cell types differing only in adsorption rate (10^-9^ or 10^-10^ mL/min). Initially, there is a 50-fold excess of cells with the higher adsorption rate, but there is a low rate at which each cell type is converted into the other. The phage ascends rapidly because most of the cell population has a high adsorption rate. However, bacterial densities are only mildly affected because the low-adsorption population displaces the high adsorption rate population. The result is paradoxical because the phage population ascends as if it will kill well, yet bacterial densities show only a slight depression; similar dynamics are seen in the case of a subpopulation of cells occupying spatial refuges. The model in this figure corresponds to the ‘intrinsic’ phenotypic resistance model in Bull et al. (2014). Parameters are otherwise the same as in **Figure 1**.

This point rests on the previous one and can be modeled with a simple extension of equation (1), in which the starting bacterial population contains a subpopulation with a lower but non-zero adsorption rate (**Figure 2**). When phage are first introduced to the naïve bacterial population, their dynamics are dominated by the average adsorption rate. However, as phage become common, individual bacteria with high adsorption rates are killed fastest; those with low rates are killed slower. The composition of the surviving population is quickly converted to one of bacteria with the lowest adsorption rate, and if that rate is low enough, phage may fail to control bacteria even though those bacteria are genetically sensitive (Bull et al., 2014). A genetically uniform population of bacteria may exhibit quantitative variation in the adsorption rate of individual cells, either by inhabiting protected sites inaccessible to the phage or by exhibiting quantitative ‘phenotypic’ resistance. The effects of phenotypic resistance and physical refuges are similar – both mechanisms enable a subset of bacteria to escape control by phages – but there is no physical structure associated with phenotypic resistance. This principle can also apply to infections spanning multiple tissues: phage growth can be at the expense of bacteria in some infected tissues, while the bacteria in other tissues escape control. Selection for phenotypic resistance directly parallels the selection for genetically resistant organisms, but because phenotypic resistance may be a physiological adaptation and offers only partial resistance, it may occur at much higher frequency in naïve bacterial populations and have little cost to the bacterium. Phenotypic resistance thus offers an immediate response to phage attack that has a high potential to thwart treatment.

#### Parameter values affecting treatment success are interrelated

Adsorption rate properties have obvious ramifications for treatment that can be identified from the models. Can we infer other dynamical determinants of success versus failure? Burst size and latent period certainly affect phage growth, although there are wide, biologically relevant latitudes in these parameter values that still allow treatment success in the standard models. In models of inundative or passive therapy, parameters of latent period and burst size are largely irrelevant, as the entire interaction is governed by the adsorption process. In models of active therapy however, where phage replication is required for success, extremely long latent periods or very low burst sizes can negatively affect predicted efficacy. Furthermore, a decline in one parameter may be offset by gains in the others, such that lower adsorption rates could be compensated for by increased burst sizes, and so on.

### SUMMARY: THE IMPORTANCE OF DYNAMICS AS DESCRIBED BY MODELS

Mathematical models of phage bacterial dynamics thus far fail to capture the detailed properties of *in vivo* treatment. The abstract models nonetheless help us understand dynamical properties that may apply *in vivo* – properties that may underlie phage failure. We can hypothesize that phages with greater adsorption rates, larger burst sizes and shorter latent periods will exhibit superior performance, but it is still not known how the *in vivo* environment affects these parameters. It is probably true that they are lower *in vivo* than *in vitro* measurements would suggest, but this is an area in significant need of study. The rate at which phage adsorb to bacteria is very likely to be a key factor in determining treatment success, and quantitative differences in adsorption rate can be a subtle determinant of phage success versus failure. The importance of individual aspects of phage dynamics to treatment success has not been established empirically, and we can further hypothesize that at least a qualitative understanding of those properties will aid in the selection of more effective phages. Evolution is another obvious dimension to consider, especially the evolution of bacterial escape. This is, however, a process on a longer time scale than the dynamics considered in this paper. Bacterial evolution could be an important mechanism of therapy failure and justifies a serious investigation.

## EMPIRICAL DYNAMICS

The preceding section described a ‘models forward’ approach to dynamics, in which the models have attempted to describe the mechanisms of different treatment outcomes, an approach which has had very limited success to date. Alternatively, phage dynamics can be measured *in vivo* first and then simple models could be developed to interpret the data.

### MEASURING DYNAMICS DIRECTLY

Ideally, we would like to know the bacterial and phage densities through time at all sites in the host. In reality, our options are not nearly this sophisticated, but they are continually improving. Various methods for *in vivo* determination of dynamics may be feasible, such as time courses of bacterial and phage numbers (Smith and Huggins, 1982; Weld et al., 2004; Scott et al., 2007; Hurley et al., 2008; El-Shibiny et al., 2009); new imaging methods allow the partial study of dynamics non-invasively (Contag, 2008; Barman et al., 2011; Henry et al., 2013). Alternatively and more simply, short-term, whole-organism phage growth rate may be measured (Bull et al., 2012). In all these methods, one is observing the dynamical outcomes that would be predicted by an appropriately parameterized model but doing so without the underlying model. This approach is especially practical for chronic infections where bacterial and phage counts can be monitored non-invasively, as with surface and gastro-intestinal infections and environmental treatments. It does not necessarily provide the same insight to mechanisms as would parameterization of a model, but with modest effort, it may be possible to fit models to the observed dynamics.

Central to any such effort is the choice of an appropriate dynamic property. Maximum phage growth rate, maximum bacterial density, total bacteria lysed, phage fecundity per infection event, and countless others are possible. However, it is not yet clear which particular properties are most relevant in any given infection system. For example, Bull et al. (2012) used 6 h dynamics to infer that each of two types of phages possessed dynamic properties suitable for numerically overwhelming the bacterial population, yet one of the phages failed to control the infection, suggesting that, in this system at least, this dynamic property (bulk phage replication) was not a determinant of treatment success.

Once a dynamic property has been chosen, the empirical data may be used in at least two different ways. As with the example given immediately above, the dynamic data can be used quantitatively, to decide whether a dynamic process or outcome is compatible with the data. Here, the data are in essence being fit to a perhaps crude but quantitative model. A second use is comparative and requires that multiple phages have been compared both for dynamics and for treatment success. One may then consider whether dynamical properties correlate with treatment success. Here, the data are not being fit to any dynamical process, only compared internally, among different phages. Such a comparison might show, for example, that phages with faster *in vivo* growth rates are superior in treatment. This comparison does not establish that growth rate is the cause of success or failure (e.g., Bull et al., 2010) or even that the growth rate of any phage is dynamically compatible with controlling the infection. But if such a comparison showed that phages with slower *in vivo* growth rate were superior in treatment, it could be concluded that this dynamical superiority was not the cause of treatment success.

If dynamics over time are not attainable, bacterial and phage counts at a single time after treatment may be informative, provided the time of assay is chosen appropriately. An approach as simple as merely dichotomizing the densities into ‘high’ and ‘low’ categories may yield considerable insight to phage quality and to the possible causes of failure (**Table 3**).

**Table 3:** Interpretation of bacterial and phage counts at a single time point after treatment.

Phage density	Bacterial density	Implication
High	High	Low adsorption rate or partial bacterial refuge, poor control of bacteria. Gives insight to failure
High	Low	Not sustainable – a possible transitory step following successful control of bacteria, before phage densities have equilibrated
Low	High	Phage unable to replicate adequately; possible genetic resistance of bacteria or inaccessible refuge
Low	Low	Treatment success


The value of counts for discriminating phages may depend heavily on the details obtained in the assays. Tissue specific counts will be more useful than whole body counts. Prior studies notwithstanding, this approach to phage therapy remains largely undeveloped as a tool for understanding success versus failure.

### COMPETITIONS: A CONVENIENT MEASURE OF RELATIVE DYNAMICS

Use of multiple phages in a treatment will nearly always result in some phages outgrowing others, provided that some of them do indeed grow. Changes in the abundances of the different phages provides a relative measure of phage dynamics that may be used to infer which phages perform best. Competitions are in fact simple dynamical assays measured on a relative scale. This method can be used on a potentially large set of inoculated phages as a way of sorting out the ‘best’ ones, reducing the number of trials that must be conducted. The determination of competitive ability is now greatly facilitated by the availability of inexpensive quantitative PCR and DNA sequencing technologies. The main drawback of competitions is that interactions among phages may alter the dynamics so that the relative growth rates do not reflect growth rates in isolation (Adams, 1959; Bull et al., 2006, 2012). This caution notwithstanding, the method is easy and could lead to useful insights. Furthermore, the competitive dynamics may reveal interactions that can be useful for or inimical to treatment when cocktails are applied.

### SUMMARY OF EMPIRICAL DYNAMICS

Although mathematical models do not capture many *in vivo* realities, dynamics may be measured empirically and give insight to mechanisms underlying phage treatment. The major limitations to this approach are largely technical, requiring the ability to observe the phage and their hosts at high spatial and temporal resolutions. Even when the dynamics can be measured, it is still not trivial to establish how dynamics contribute to treatment outcome: phages with superior dynamics may be superior at treatment for reasons other than dynamics. Despite these unknowns, the use of dynamics to understand outcomes is worthy of investigation.

## INDIRECT EFFECTS: ALTERNATIVE PATHS TO EFFICACY

We have raised the possibility that phage dynamical properties may not be the main determinant of treatment success. Yet aside from the principles listed in **Table 1**, alternatives to phage success have not been offered. Indirect effects of phages provide one such alternative. Indirect effects are phage-caused modifications of the environment that alter bacterial dynamics or alter the host response; they can be considered effects of a phage that extend beyond burst size, lysis time, and adsorption rate. We offer three diverse examples.

### THE IMMUNE RESPONSE AFFECTS OUTCOME

As a rule, phage do not fully clear large bacterial populations on their own, they merely reduce bacterial numbers. The complete clearing of an infection by phage must thus rest with the immune system, and phages may differ in how they affect immunity. Phage-encoded depolymerase enzymes can be sufficient to cure an infection (Avery and Dubos, 1931; Francis et al., 1934; Sutherland, 1995; Mushtaq et al., 2004); such enzymes presumably expose bacteria to constitutive immune defenses by stripping away capsules and other protective layers on the bacterial surface (Mushtaq et al., 2004; Scholl et al., 2005). Depolymerases are observed as components of phage tailspike assemblies, and a significant proportion of these tailspikes are thought to be released unassembled as free enzyme upon cell lysis. Thus phages with those enzymes both kill bacteria and help the immune system kill the bacteria that phage miss. This indirect effect improves treatment.

A second suggestion of the importance of immunity comes from a study showing that the physical lysis of bacteria worsens treatment outcome, presumably because lysis releases endotoxins (Hagens et al., 2004; Matsuda et al., 2005; Paul et al., 2011). In this case, the indirect effect of the phage — the release of endotoxins — hampers treatment.

Finally, biofilms may present barriers to phage access; the exopolysaccharide matrix (EPS) is considered to be one such barrier. Phages that produce enzymes degrading the EPS are thought to impart improved biofilm clearing over phages lacking such enzymes because the enzymes destroy the physical structure of the biofilm (Hughes et al., 1998; Azeredo and Sutherland, 2008). Futhermore, phages may even be engineered to express biofilm-degrading enzymes that improve biofilm clearing (Lu and Collins, 2007). Although these enzymes may have the effect of exposing greater numbers of bacteria to phage killing, enzyme has been shown to be sufficient for biofilm clearing in the absence of phage (Schmerer et al., 2014), thus indicating an indirect effect of the engineered phage. This example parallels that above of capsular depolymerases enhancing immune clearing of bacteria.

### DIAGNOSING INDIRECT EFFECTS

Indirect effects present a distinct contrast to the model that treatment success stems from phage dynamics. They thus offer an important avenue for study, but it is not trivial to demonstrate indirect effects, because they will usually be confounded by dynamics. In the three examples cited above, indirect effects were evident because a manipulation enabled treatments that changed the balance between dynamics and indirect effects (a non-lysing phage that improved survival, treatment with an enzyme alone improved the outcome). Genome engineering and the generation of isogenic phage variants will provide one fruitful avenue to explore a wide range of possible direct and indirect effects.

### SUMMARY OF INDIRECT EFFECTS

There are sporadic demonstrations in which treatment success does not result directly from rampant phage killing, and these indirect effects offer a distinct alternative to dynamics as the basis of phage therapy success. There is already evidence that they are important in some systems. The degree to which they influence treatment success will have a major effect on the rational choice of phages for treatment.

## COMPARATIVE METHODS CAN IDENTIFY CAUSES OF PHAGE SUCCESS

The focus of this paper has been the ‘Central Question’ on the importance of phage dynamics to therapeutic success. On the surface, the path to answering this question would seem to be the direct approach of quantifying phage dynamics and determining whether they can explain treatment outcome. This direct approach is difficult at least because the cause–effect relationships are not obvious from dynamics alone, and many types of experimental manipulations are required to establish whether quantitative properties of growth *per se* affect treatment outcome.

An alternative, powerful approach to identifying causes of treatment success is the comparative method (Pagel and Harvey, 1988; Harvey and Purvis, 1991): characteristics of phages with high success rates are compared to characteristics of phages with low success rates. Characteristics differing between the two groups become candidates for causation. The characteristics considered might be genetic or lifestyle characteristics (e.g., receptor type, DNA ejection and host-takeover mechanisms) as well as various gross phenotypes (e.g., growth rate, virion size, virion charge). Among other applications, the comparative method can be used to determine if treatment success correlates with dynamic properties.

Applications of the comparative method to phage therapy are surprisingly rare in the literature, as there are few studies meeting its minimum requirement: identification of both effective and ineffective phages for the same infection model. The classic comparative study is by Smith and Huggins (1982) using mice infected with *E. coli* O18:K1:H7. Across a set of wild phages, those requiring the bacterial capsule were invariably more effective at saving mice (at least when treatment was administered concurrently with bacteria), whereas those using a different receptor were invariably poor. In a second more recent example, a panel of *Pseudomonas aeruginosa* phages was evaluated both *in vitro* and *in vivo* in a mouse infection model (Henry et al., 2013). In the latter case, simple plaque-forming ability (measured as EOP) was found to be a poor predictor of therapeutic efficacy, while the ability of the phage to clear liquid cultures was a better but still imperfect predictor. Phages isolated from the environment on the mouse challenge strains were found to be more effective in treatment, but what specific phage attributes were key to therapeutic success remains to be determined. Differences in phage efficacy have also been reported in treatment of plant infections (Balogh et al., 2010).

The challenge with the comparative method is to identify causal characteristics, not just correlates. Causation can be inferred when there is only a single variable responsible for the correlation (when all but one variable is controlled for in the comparison). For example, in the case of Merril et al. (1996), the effective and less effective phages were isogenic except for a point mutation in the capsid protein gene, that mutation necessarily being causal. For the capsule-dependent and -independent phages of *E. coli* O18:K1:H7 in the Smith-Huggins mouse model, the endosialidase tailspike was identified as the critical determinant of treatment success because the only significant genetic difference between one pair of phages was that gene (Bull et al., 2010). Conversely, when the effective and ineffective phages differ in multiple characteristics, as in the case of Henry et al. (2013), it may not be possible to identify the crucial difference without some form of genetic manipulation to experimentally alter the phage genome. However, to assess whether dynamic properties determine success, an attainable initial step is merely to compare dynamic qualities between the two classes of phages.

### SUMMARY OF COMPARATIVE STUDIES

The comparative method offers a fast approach to identifying correlates of treatment success. There are yet few applications of the comparative method to identify the basis of phage success versus failure, no doubt because there are few systems that have identified parallel sets of phages with high and low efficacy. This is perhaps the most promising avenue to pursue in resolving the basis of treatment success.

## FUTURE DIRECTIONS

Phage therapy need not be confined to the practices used in the past. Phages may be combined with other treatments or may be engineered with properties that do not occur naturally.

### ADSORPTION RATE AND INSIGHTS TO IMPROVED THERAPY

The qualitative behaviors inferred from the models provide guidance toward possible mechanisms of phage failure and thus toward ways to choose better phages. They first suggest that it is not merely sufficient to isolate phages that form plaques on the target bacterium or grow well when first introduced into its populations: those criteria do not avoid phages that are susceptible to escape by cryptic bacterial mechanisms. These principles do suggest courses of action: if phage do not control bacteria *in vivo*, escaping bacteria should be checked for genetic resistance to the phages. Lack of genetic resistance suggests an access problem – phenotypic resistance or refuges. Some kinds of phenotypic resistance may be overcome by use of phage that are able to bypass this phenotype. Depending on their nature, physical refuges may pose a problem to many types of phage, and may be circumvented by use of more persistent or better-penetrating phage strains, or by using different routes of administration. Finally, because the physiological state of the bacterium can strongly affect subsequent phage dynamics, there must be a greater understanding of the *in vivo* status of the pathogen and how this affects the ability of the phage to infect and replicate in its host. Ultimately, there is a need to identify classes of bacterial escape mechanisms to devise strategies to thwart bacterial escape. We might hope that *in vitro* assays of phage-bacterial dynamics can be used to identify phages worthy of *in vivo* testing, but it may also be that useful identifiers of phage efficacy await the generation of a large database of correlations between *in vitro* and *in vivo* dynamics.

Our current understanding leads us to suggest that there may be classes of bacterial surface molecules that, when used by phages for adsorption, are not prone to bacterial escape by refuges or phenotypic resistance. Capsules constitute an obvious target fulfilling this criterion, because capsules are typically dense enough that the bacterium may not be able to simultaneously reduce adsorption by a phage targeting the capsule and also avoid immune attack. We hypothesize that it should be possible to identify types of bacterial surface molecules that avoid non-genetic bacterial escape and to then use that knowledge to choose phages *a priori* for therapy. It is too early to know if that knowledge can be generated from *in vitro* data. However, this hypothesis is consistent with the common observation that capsular depolymerases improve phage treatment success both *in vivo* and in biofilms (Sutherland, 1995; Azeredo and Sutherland, 2008). A systematic study of phage receptors and quantitative bacterial escape may reveal several types of phage targets that avoid or reduce bacterial escape, with capsules being just one. A first step toward this goal is to study dynamics *in vitro* (*in vivo*, if possible) and observe the details of bacterial decay and recovery in the presence of phage. Phages that have better properties at depressing bacterial densities and maintaining low densities should be candidates for application.

### COMBINATIONS OF PHAGES AND ANTIBIOTICS

A long-touted benefit of phages is that they can grow on antibiotic resistant strains of bacteria: the genetic bases of drug resistance rarely prevent phage growth. However, a less obvious tie between phages and antibiotics is that they may complement each other in treatment, both being more effective in combination than either is alone. A practical utility of studying phage therapy during antibiotic treatment is that clinical trials often require the testing of new treatments in a background of standard treatment — antibiotics.

Even when a bacterium is sensitive to an antibiotic and a phage, treatment with either alone often results in a subset of bacteria that avoid killing. Several reports now reveal greater bacterial killing when phage and antibiotics are used in combination than with treatment by either alone (Huff et al., 2004; Bedi et al., 2009; Verma et al., 2009, 2010; Rahman et al., 2011; Chhibber et al., 2013; Viertel et al., 2014). This ‘synergy’ is typically quantitative, not absolute, so many bacteria escape the combined treatments. The dynamical benefit from combined treatment has not been investigated; it must ultimately be that each agent kills subpopulations escaping the other agent. Understanding and comparing the basis of escape with single and double treatment should contribute to understanding phage therapy in the absence of drugs. Merely identifying whether the best phages in combination with drugs are also the best phages when used alone should lead toward an understanding the basis of phage success.

### ENGINEERED PHAGES

Advances in DNA synthesis and genomics will enable the facile genetic engineering of phages. Most importantly, phages may now be endowed with genes and phenotypes that would never be observed in nature. Some of these modifications may be of diagnostic value (e.g., Schofield et al., 2013; Peng et al., 2014), others may be useful in treatment (e.g., Lu and Collins, 2007). These modifications include using phages as chemical delivery agents, using phages to change the bacterial genome, expanding phage host range, blocking phage reproduction, encoding phages with enzymes that degrade extracellular matrices, and others (e.g., Westwater et al., 2003; Lu and Collins, 2007, 2009; reviewed in Viertel et al., 2014). The dynamical impacts may be to improve phage growth, create/alter indirect effects, or even to modify bacterial competitiveness. As noted above, phage engineering may greatly advance the study of both dynamics and indirect effects, as these engineering methods should also provide tools for generating the defined isogenic phage variants that can be used for comparative *in vivo* or *in vitro* studies.

Success is not assured by the mere engineering of a desired phenotype. Success will also rest on dynamical issues—the engineered phenotype must be of sufficient magnitude and the phage density must attain sufficient levels to impact treatment. Dynamic analysis of the therapeutic failure of an engineered phage may thus suggest ways to render it successful. However, a further impediment to the success of engineered phages is short-term evolution: phage reproduction during treatment may reverse the engineering (Gladstone et al., 2012). Phages engineered to provide indirect effects that do not benefit phage growth will be especially prone to evolutionary reversal, but engineering that benefits phage population dynamics without specifically benefitting the engineered genome is also prone to reversal (Gladstone et al., 2012). Fortunately, there is a simple solution to evolutionary reversal: repeated inoculations with high doses of the engineered phage should maintain moderate levels of the engineered phage despite opposing evolution.

The potential of engineering extends well outside traditional bounds of phage therapy. Temperate phages have been engineered to render drug-resistant bacteria sensitive to antibiotics without killing them (Edgar et al., 2012). This approach may at first sight seem useless—the phage might just as well kill the bacteria as modify it. But the ecological effect of changing the bacterial genome is to alter competition with drug-resistant bacteria in the external environment, thus reducing the frequency of drug resistance before an infection starts. The success of such an intervention is obviously sensitive to many ecological and dynamical details, but the work at least serves as an inspiration for many non-traditional uses of engineered phages – and further highlights the possible relevance of dynamics to success.

## RECOMMENDATIONS

We suggest that the science of phage therapy will be advanced by discovering principles that improve success, and the hope is that principles from one system will apply to others. We offer the following recommendations that highlight some of the preceding points.

### COMPARE THE GOOD AND THE BAD

Contrast therapeutically effective and ineffective phages to identify the causes of treatment success and/or failure. To apply the comparative method, different phages must have been tested in the same infection model, and some phages must yield better treatment outcomes than the others. This approach can be used regardless of the mechanism responsible for treatment success/failure. The comparative approach has identified capsular depolymerases as the basis of treatment success in one system; rather than relying on the natural occurrence of convenient phage pairs, the further development of genetic tools to create targeted gene deletions or replacements in virulent phage will facilitate direct comparisons of isogenic phage pairs.

### EXPLORE AND CONSIDER DYNAMICS

A phage’s ability to grow *in vivo* is central to our understanding of treatment outcome. Dynamics need not be the determinants of success, but the relative contribution of dynamics to treatment success needs to be understood. At present, it is practical to study dynamics only qualitatively or in competitions, but even crude assessments may pay off. Furthermore, study of dynamics can be coupled with the study of indirect effects to assess whether dynamics is the key determinant of success.

### UNDERSTAND BACTERIAL ESCAPE

Phage killing will often be only partial, with some bacteria escaping. In turn, treatment outcome can depend on the quantitative details of escape. Mutational resistance is a commonly cited mechanism of bacterial escape but there are several other, less-understood mechanisms such as transient phenotypic changes and physical refuges that warrant consideration. Understanding mechanisms of bacterial escape may thus prove valuable in choosing appropriate phages, for example by avoiding types of receptors that are prone to variation in the bacterial population or by using phages that degrade exopolysaccharide matrices. Pathogen escape in spatial refuges may be circumvented by altering the route of phage administration or coupling phage treatments with surgical procedures such as debridement. The model system of infection is especially relevant here, because the details of escape may be highly sensitive to the pathogen, host, and site of infection.

### USE RELEVANT MODELS OF INFECTIONS

The empirical systems used to evaluate phages must mirror the applications in which phages will be used. It is obviously convenient to evaluate phages *in vitro*, but good phage performance in culture medium can be a poor predictor of phage success *in vivo* (e.g., Balogh et al., 2010; Tsonos et al., 2013b). *In vitro* relevance may be enhanced with a system that mimics the *in vivo* environment closely: the culture of *Staphylococcus aureus* in a system of native milk proteins showed strong inhibition of phage adsorption, even though the phage was able to aggressively control the bacteria in broth culture (O’Flaherty et al., 2005; Gill et al., 2006). Models that attempt to evaluate phages under more realistic conditions may include *ex vivo* media (serum, blood, lymph, etc.), tissue culture or biofilm-promoting conditions. Even animal models may be misleading because they can behave differently than natural infections found in patients (Bull et al., 2002; van der Worp et al., 2010). Studies of phage dynamics in agricultural animal systems possesses a distinct benefit here, as in these cases the animal model is also the ultimate treatment target and the experimental observations will be more directly related to practical application.

### MODELS

A new generation of models is needed, especially for dealing with spatial heterogeneities. Parameterization of such models will not likely be possible *in vivo*, but such models will nonetheless be useful in identifying types of complexities that thwart treatment. Once identified, those processes critical to dynamics may become tractable for empirical study. At present in the absence of such models, we have little idea what to look for. Such models may need to be computational, as the analytic approximations required to solve spatially structured equations do not lend themselves to spatially varying parameter values.

## CONCLUSION

The field of phage therapy is ripe for approaches that explore mechanisms underlying treatment success versus failure. We still do not understand which phage properties are important to success, whether those properties generalize across infection, and whether dynamical superiority translates into treatment superiority. At the optimistic end of the spectrum, we may hope that the only important consideration for treatment is to use a phage whose host range includes the infecting bacterium. The pessimistic end of the spectrum is that no phages will work on some bacteria. Between these extremes, there is a wealth of opportunity to discover how to make phage therapy a success by the judicious exploitation of *in vitro* characterization, genomics, and experiments in appropriately designed animal models.

## Conflict of Interest Statement

The authors declare that the research was conducted in the absence of any commercial or financial relationships that could be construed as a potential conflict of interest.
